# Characterization of two new *C. briggsae* multivulva genes

**DOI:** 10.17912/micropub.biology.000859

**Published:** 2023-06-12

**Authors:** Nikita Jhaveri, Bhagwati Gupta

**Affiliations:** 1 Biology, McMaster University, Hamilton, Ontario, Canada

## Abstract

The nematode *C. briggsae* is an excellent genetic model for comparative and evolutionary studies involving its well-known cousin *C. elegans* . The vulval system in these two species has been used extensively to investigate genes and pathways involved in cell proliferation and cell differentiation. Here we report initial characterization of two *C. briggsae* multivulva (Muv) mutants, *Cbr-lin(bh1)* and *Cbr-lin(bh3)* .

**Figure 1. Muv penetrance, % induction and polymorphism mapping of Cbr-lin(bh1) and Cbr-lin(bh3):**
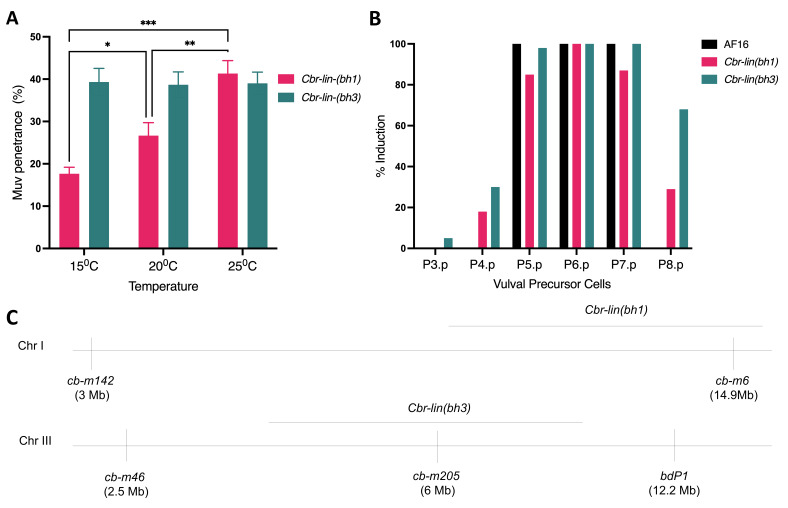
A. Muv penetrance was examined at 15℃, 20℃ and 25℃. Statistical test was carried out using one-way ANOVA with Bonferroni's multiple comparisons test (n= 40-50 from a total of 3 batches for each genotype and temperature). Muv penetrance for *Cbr-lin(bh1)* : 18% +/- 2% (15℃), 27% +/- 3% (20℃), and 41% +/- 3% (25℃); and for *Cbr-lin(bh3)* : 38% +/- 3% (15℃), 38% +/- 2% (20℃), and 40% +/- 4% (25℃). Asterisk (*) indicate statistically significant *p* values (*: *p* < 0.05, **: *p* < 0.01, ***: *p* < 0.001). B. VPC induction pattern of *Cbr-lin(bh1)* and *Cbr-lin(bh3)* animals at 20℃ *.* C. *Cbr-lin(bh1)* showed linkage to the indel *cb-m6* but not to *cb-m142* , suggesting its location on the right arm of chromosome I. *Cbr-lin(bh3)* was linked to *cb-m205* but not to *cb-m46* and *bdP1* , which placed the gene roughly to the middle of chromosome III. The physical locations of the markers are in parentheses.

## Description

Nematodes of the genus *Caenorhabditis* are routinely used to investigate genes, genetic processes, and conserved signaling pathways. The vulva, a reproductive organ in *C. elegans* and *C. briggsae* , is often utilized for comparative analysis due to its invariant cell lineage and well-defined cell fates (Gupta et al. 2007; Sternberg, 2005) . Previous studies have shown that though the morphology of the vulva is similar in these nematodes, there are substantial differences in the underlying genetic networks (see, for example, Chamberlin et al., 2020 and Sharanaya et al., 2012). Identifying and characterizing the genes that affect vulva formation in *C. briggsae* should enable further studies of evolutionary differences between the two species.

The multivulva (Muv) mutants, *Cbr-lin(bh1)* and *Cbr-lin(bh3)* , were recovered in a forward genetic screen in our lab. The phenotype of *Cbr-lin(bh1)* , but not *Cbr-lin(bh3),* was found to be heat sensitive such that the penetrance of the *Cbr-lin(bh1)* Muv phenotype positively correlated with temperature ( Figure 1A ). There was no maternal contribution to vulval precursor cell (VPC) induction (4.9% Muv in the progeny of *Cbr-lin(bh1)* heterozygous mothers, n = 309, vs. expected ~7%, i.e., one-quarter of 27% Muv; and 9.3% Muv in the progeny of *Cbr-lin(bh3)* heterozygous mothers, n = 313, vs. expected ~10%, i.e., one-quarter of 38% Muv). Nomarski microscopy revealed that P3.p, P4.p and P8.p VPCs were ectopically induced in mutant animals ( Figure 1B ). Two cell fate markers, *Cbr-egl-17::GFP* ( mfIs5 , 2 ^0^ marker) and *Cbr-zmp-1::GFP* ( mfIs8 , 1 ^0^ marker) (Félix 2007), were used to examine the VPC fates. While ectopically induced VPCs in *Cbr-lin(bh3)* animals adopted both 1 ^0^ and 2 ^0^ fates (1 ^0^ : 19.6%, n = 158 and 2 ^0^ : 97.9%, n = 190 instances of induced P3.p, P4.p and P8.p), only 2 ^0^ fates were observed in *Cbr-lin(bh1)* animals (1 ^0^ : 0%, n = 104 and 2 ^0^ : 96.4%, n = 167 instances of induced P3.p, P4.p and P8.p).

We also observed that *Cbr-lin(bh1)* animals grow slower, taking almost 20 hours more than the wild-type AF16 to reach adulthood. The *Cbr-lin(bh3)* strain showed no such growth delay. Indel-based polymorphism mapping (Koboldt et al., 2010) assigned *Cbr-lin(bh1)* to the right arm of chromosome I and *Cbr-lin(bh3)* roughly to the center of chromosome III ( Figure 1C ). Complementation tests of *Cbr-lin(bh1)* with existing chromosome I Muv mutants, *Cbr-spr-4(gu163)* and *Cbr-pry-1(sy5353)* , suggested that it represents a distinct gene (0% Muv, n = 117 F1 heterozygotes and 0% Muv, n = 28 F1 heterozygotes; respectively).

## Methods

Material and methods

Worm culture maintenance and genetic screen

All strains were grown on the Nematode Growth Medium (NGM) plates seeded with OP50 bacteria (Brenner 1974) , and maintained at 20℃ unless otherwise specified. AF16 worms were mutagenized by feeding them with 25 mM EMS for four hours. Individual P0s were allowed to grow on plates and F2 animals were screened for a Muv phenotype. Candidates were isolated and phenotype was confirmed in successive generations. *Cbr-lin(bh1)* and *Cbr-lin(bh3)* strains were outcrossed 2x before characterizing their phenotypes. The strains used in this study are listed in the following table.

**Table:** 

Strain name	Genotype	Source
AF16	Wild-type	CGC
CM2342	*Cbr-spr-4 (gu163); mfIs5 [Cbr-egl-17::GFP; Cel-myo-2::GFP] *	Chamberlin lab
DY1	*Cbr-lin(bh1)*	Gupta lab
DY250	*Cbr-pry-1 ( sy5353 ) *	Gupta lab
DY3	*Cbr-lin(bh3)*	Gupta lab
DY632	* Cbr-lin(bh1); mfIs5 [Cbr-egl-17::GFP; Cel-myo-2::GFP] *	Gupta lab
HK104	Wild-type	CGC
JU613	mfIs8 [ *Cbr-zmp-1::GFP; Cel-myo-2::GFP* ]	Felix lab

Polymorphism mapping

Mapping of *Cbr-lin(bh1)* and *Cbr-lin(bh3)* was carried out using medium indels (Koboldt et al. 2010) . Briefly, mutants were crossed to HK104 males. Muv animals were selected in the F2 generation and were pooled for PCR analysis. See Sharanaya et al., (2015) for a mapping scheme and the protocol. The details of indel polymorphism markers and primers are mentioned in the table below. PCR products were analyzed on 1% agarose gel and the linkage was determined based on qualitative assessment of the intensity of AF16 and HK104 bands.

**Table:** 

**Chr #**	**Indel-based polymorphism marker**	**Primer pair**	**Annealing temperature**
Chr I	*cb-m142* (250 bp)	GL588/GL589	50℃
Chr I	*cb-m6* (100 bp)	GL905/GL906	50℃
Chr III	*cb-m46* (270 bp)	GL1160/GL1161	55℃
Chr III	*cb-m205* (300 bp)	GL1143/GL1144	53℃
Chr III	*bdP1* (200 bp)	GL1162/GL1163	58℃

Muv penetrance and VPC induction analysis

Animals were grown for at least three generations at a given temperature (15℃, 20℃ or 25℃) before scoring the phenotype. Each genotype was scored in triplicates. In each case 40 - 50 worms were examined under a Nikon Eclipse 80i Nomarski microscope.

L4 stage worms were analyzed for VPC induction by mounting them on agar pads containing 1mM Sodium Azide. 50 - 60 animals, pooled from three batches, were examined for each genotype at 20℃.
